# Meeting report for SIGS1: First Conference of the Standards in Genomic Sciences eJournal

**DOI:** 10.4056/sigs.328

**Published:** 2009-07-20

**Authors:** Oranmiyan W. Nelson, Scott H. Harrison, George M. Garrity

**Affiliations:** 1 Department of Microbiology and Molecular Genetics, Michigan State University, East Lansing, Michigan, USA

## Introduction

SIGS1 was convened on March 13-14, 2009 at Michigan State University, East Lansing, Michigan, USA to review progress toward publication of the first issue of the Standards in Genomic Sciences (SIGS) eJournal. In attendance were members of the advisory and founding editorial board. SIGS was conceived to fill a growing need: to provide a genome-centric venue for publication of the increasing volume of genomic and metagenomic data that is now without a formal publication in the scientific, technical and medical (STM) literature in a standards compliant manner. A central goal of SIGS is to apply concepts developed by the Genomic Standards Consortium, such as the MIGS checklist (Minimum Information about a Genome Sequence), to published articles so as to make them more accessible to both human and machine readers [1]. To accomplish these goals, it is important that all of the metadata associated with the source organism, the sequencing methodologies, genome assembly, and genome annotation methods be reported in a consistent manner and become a part of the formal published record.

## News from the community

The meeting opened with introductions and an exploration of areas of common interest by members of the Genomic Standards Consortium (GSC) and the MSU genomics community. In attendance were faculty and staff members, postdoctoral fellows, and graduate students from the MSU Microbiology and Molecular Genetics Department and the Center for Microbial Ecology. Several additional members of the GSC participated via videoconferencing.

Estelle McGroarty, Assistant Vice President for Research and Graduate Studies, welcomed attendees and discussed the scientific growth and achievements of Michigan State University (MSU), and the nature of the institution as a land-grant university. She was followed by George Garrity (MSU), who gave an introduction to SIGS, describing the technical tasks of the editorial office, article types, interlinking with other content, indexing, archiving and editorial workflow. He pointed out that much of the groundwork was already completed, but that key tasks remained prior to publication of the first issue of the journal.

An update from the Genomic Standards Consortium was provided by Dawn Field (NERC Centre for Ecology and Hydrology, UK), who described interrelated organizations and initiatives including the International Nucleotide Sequence Database Consortium (INSDC), Minimum Information About a Proteomics Experiment (MIAPE) and others. She also described the overall goals for capturing contextual data, initiatives for adoption of the MIGS specification by the broad ‘omics community, and recent meetings of the GSC.

**Figure 1:**
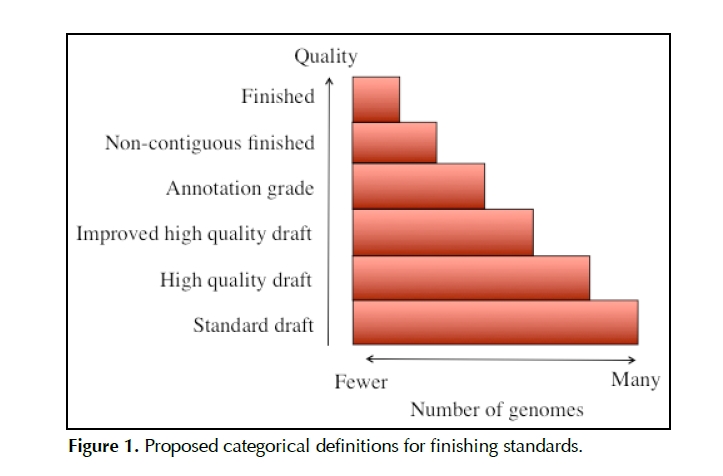
Proposed categorical definitions for finishing standards.

Patrick Chain (DOE Joint Genome Institute; JGI) presented work on standards for genomic sequencing pipelines, with an emphasis on categorical definitions of sequence quality, especially in reference to the finishing phase. Because of the technical difficulties involved in filling sequencing gaps, identifying artificially extended repeats, and resolving other ambiguities produced by various sequencing technologies, gradations exist between sequenced genomes that make a simple set of terms such as *complete* and *incomplete,* or *finished* and *draft*, inadequate. He presented work from the genomic sequencing community for other proposed categorical definitions of finishing standards such as "high quality draft", "improved high quality draft", "annotation grade", "non-contiguous finished", and "finished", and he projected the relative numbers of genomes associated with each category (see Figure 1).

Chain was followed by presentations of metagenomic and genomic biology projects at MSU. Rob Britton gave an update on efforts made to bolster genomics education at the undergraduate level. He has incorporated the Integrated Microbial Genomes Annotation Collaboration Tool (IMG-ACT) into projects assigned to students in his microbial genomics courses. By using IMG-ACT, students are given first-hand experience at extracting usable information from complex data by annotating a suite of provided microbial genes. As the field of genomics accelerates, earlier exposure of students to genome sequencing and annotation will be essential. Tom Schmidt gave a review of metagenomic studies on the effects of deforestation on microbial community structure. Schmidt described the Long Term Ecological Research (LTER) Network, and the use of molecular surveys of key functional genes to measure the relative abundance of bacterial species in microbial communities [2]. Of particular interest is the presence of duplicates within draft level metagenomic data that exist as artifacts of the MG-RAST automated sequencing technology. Although artificial duplicates can greatly skew measurement of gene abundance, they can be easily removed by selecting the appropriate parameters in MG-RAST.

Paul Gilna (UCSD) presented news from the CAMERA project (Community Cyberinfrastructure for Advanced Marine Microbial Ecology Research and Analysis), and emphasized the importance of intact background information on organisms as expressed by the policies of the Moore Foundation which require the inclusion of metadata in genome sequence submissions [3]. Gilna also described challenges of using tools like BLAST to conduct large-scale queries against genome databases. Lynette Hirschmann (MITRE) discussed metadata capture, especially in terms of: machine-readable tables based on MIGS, the goal of establishing MIGS-based data reports for every sequenced organism, the Habitat Lite vocabulary for reporting the collection locale of an organism, and the ontology-based link of Habitat Lite to the Environmental Ontology (EnvO) system [4]. Peter Sterk (Sanger) discussed the development of curation resources for furthering the MIGS standard for genome reporting. Maintaining the integrity of metadata as it is reported (if it exists in the first place) is a crucial and time-consuming task that requires greater resources as the number of sequenced genomes climbs well into the thousands.

A summary of recent developments in GSCs standards was provided by Lynn Schriml (University of Maryland, Institute for Genome Sciences). This included a discussion of ontology development (EnvO), the Gaz geographic location vocabulary and the Genomic Rosetta Stone (GRS) project. These efforts are aimed at making genomic databases more integrated and usable through the mapping of terms and identifiers, and fostering the development of standards within the genomics community

Nikos Kyrpides (JGI) reported on the rate of growth in the number of sequenced genomes, which is now projected to reach 2500 finished sequences by 2010, even according to conservative estimates. Despite the large quantity of data, there is an over-representation of Proteobacteria among currently sequenced genomes, greatly skewing our view of the microbial world. In response to this gap in knowledge, the JGI has launched the Genomic Encyclopedia of Bacteria and Archaea project (GEBA) in cooperation with the Deutsche Sammlung von Mikroorganismen und Zellkulturen (DSMZ) to enrich the view of microbial diversity by sequencing and reporting on 100 genomes of broadly distributed phyla. Genomic data from the GEBA project are a promising source of articles for SIGS. Kyrpides described various enhancements to GEBA-related articles intended for submission to SIGS such as electron micrographic pictures of microorganisms and metabolic pathway diagrams.

Sam Angiuoli of the University of Maryland discussed the necessity of efficient standardized reporting of the protocols used in annotation pipelines because of the wide variety of methodologies used to sequence and annotate genomes. The information included in SOPs and the style of reporting varies greatly between institutions. In addition, the location of SOPs on the web are not centralized [5]. For these reasons, a central location for SOPs that are reported in a uniform fashion is of great need. Such an SOP repository would foster review of the methods, parameters and assumptions used by various centers and enhance the reproducibility of genome annotations. To this end, the importance of SIGS as a place to report SOPs was discussed.

## Content for the journal

Scott Harrison (MSU) reviewed the history of the relationship between bioinformatics and published forms of data [6]. This included a brief survey of the increasing number of sequenced genomes lacking a corresponding publication, and a discussion of the non-uniform coverage of topics in genome sequence-related publications. Harrison also presented a summary of article types for SIGS. Primary article types for the first several issues of publication are white papers, meeting reports, short genome reports and standard operating procedures. An important block of articles being queued for submission consists of the GEBA genomes, as well as a number of other genomes of taxonomic type strains of bacteria that are already in the public domain, but do not yet have a corresponding publication.

The SIGS editorial approach is designed to lessen error by ensuring peer review-based verification of reported data and its conformity with MIGS. While the key focus of SIGS is to tightly integrate and link authored content with the MIGS specification, other article types may aid in the process of channeling feedback from investigators to the standards community. The full range of article types being considered for SIGS also includes reviews and commentaries, data policies, and other gray literature that are relevant to genomic sciences, but have been absent from the scholarly literature [7]. Additional advantages to publication in SIGS are considered to be: linking to previously published standards-compliant content and standard operating procedures, and relinking to article content based on updates to the standard.

**The anatomy of a short genome report consists of:**
- *front matter* – title, authors and keywords;
- *description of the organism* – phylogenetic tree, photomicrographs or electronphotomicrographs and a table on the general classification and features of the organism;
- *genome sequencing project information* – a table and series of article subsections on sequencing, assembly and annotation;
- *genome properties* – counts of genome size and genes, and other related figures such as genome maps and COG-based comparisons; and
- *references* – with a mention of the customized bibliography style developed for the journal.

Different approaches for collecting MIGS-based annotations were discussed including use of the SIGS document template, customized checklists and form fields within the editorial workflow. Externally hosted XML-based database entry forms with pre-populated selection lists were also considered. While tables and figures are commonly contained within the manuscript body, it was recommended that these materials be submitted separately. Tables reporting the general features and phenotype of the organism, and genome sequencing project information were discussed. In particular, deliberation was held as to how the tables should be remodeled to follow the scope and organization of the MIGS specification. It was concluded that columns with MIGS IDs be added to the first two tables of a short genome report, and that the general order and contents of the first two tables remain the same. As a separate item to the short genome report, the MIGS-based data record for a genome will be also incorporated, although availability of data may be limited for older data sets derived from genomes already in the public domain. There was also additional discussion concerning the GCDML (Genomic Contextual Data Markup Language) [8] and how short reports may interoperate with GCDML-based web services that may eventually facilitate the capture, exchange and comparison of a large amount of data.

## Operation and publishing

**Figure 2:**
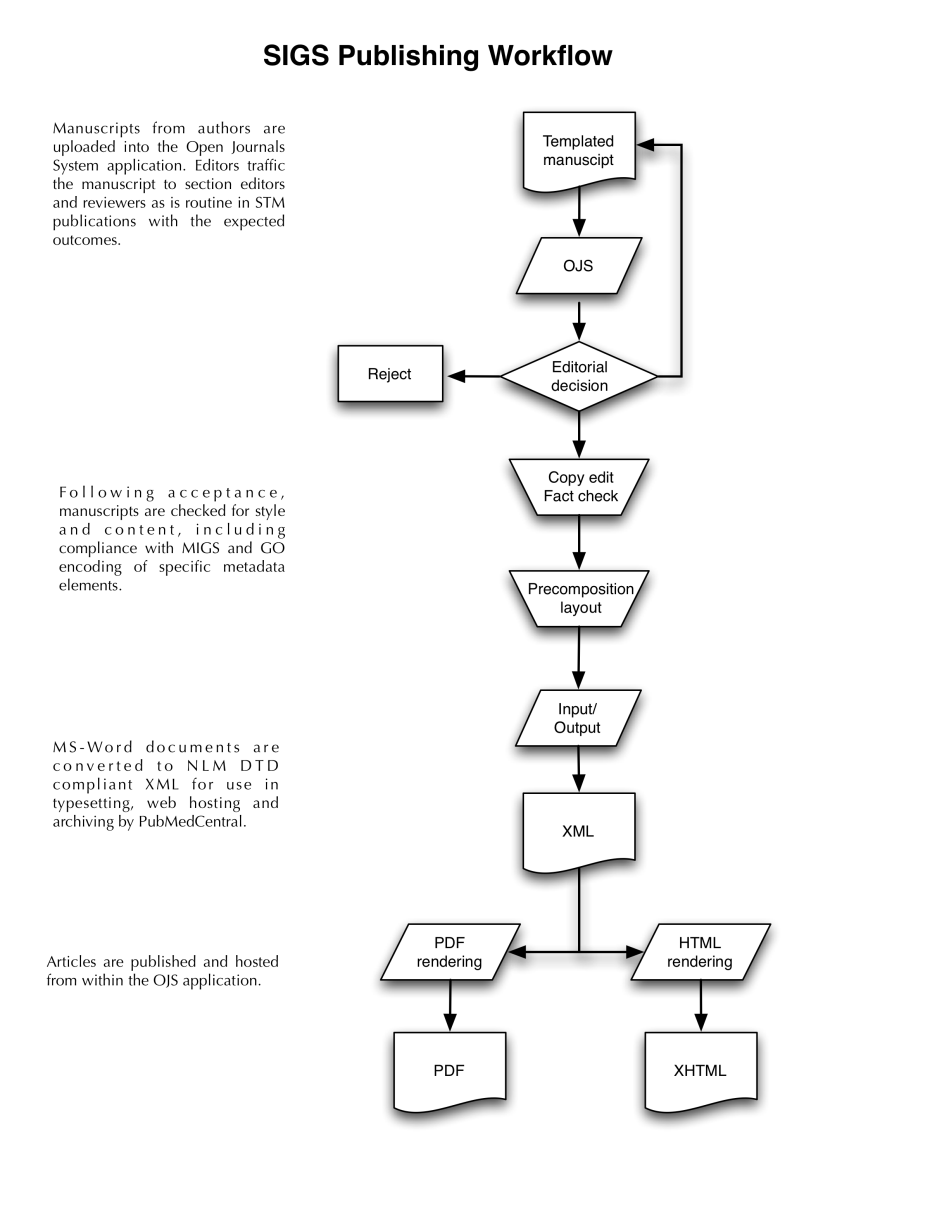
Editorial workflow.

SIGS uses Open Journal Systems (OJS), an open source management and web-hosting software application. Editing and peer-review in the SIGS workflow is facilitated by the editorial office in coordination with sequencing centers. After completion of a manuscript and checklist, the submission enters the editorial queue. An editor selects a section editor to monitor the progress of the document through the pipeline. Section editors invite reviewers based on research interest and availability. Based on feedback from reviewers, section editors inform authors as to the necessary revisions that must be made for acceptance. Revised manuscripts then enter into the publication pipeline, undergoing copyediting, layout and publication in HTML, PDF and XML based on the National Library of Medicine Document-Type Definition (NLM-DTD). Both manuscript trafficking and web-hosting of content are handled using the OJS software. SIGS has been assigned an ISSN by the National Serials Data Program (NSDP) at the Library of Congress and is also a member of CrossRef. As a CrossRef-enabled publication, every article published in SIGS will be issued a DOI, and the bibliographic data interlinked with the content of other publishers to help drive both the impact factor of the journal and the *h*-numbers of authors. The possibility of semantic interlinking through the use of NamesforLife technology was also discussed. The SIGS website and editorial workflow were presented to the audience by Harrison and Garrity, with simulation of how editors and reviewers would process a contributed article (see Figure 2).

## Summary and outcome

SIGS subscribes to the concept of open access publishing and Creative Commons licensing of content, with the majority of rights remaining with the authors. At this stage of development, authors may submit manuscripts free of charge, however this policy may change after the first six months of publishing. It was agreed that authors may be charged a modest fee in the future to help defray publication costs. Copyrighting was also discussed, but several unresolved issues remain to be further explored, including SIGS/GSC rights to update content in the future as additional data become available. The editorial board also needs to further examine the conventions and policies related to the assignment of DOIs and linking to external content to ensure persistence and compliance in the future, should all rights be assigned back to authors.

A second issue that could not be resolved was integration of GCDML into the publishing workflow. Several options were explored, including integration of GCDML into the NLM DTD, use of GCDML terms within the existing NLM tag set; incorporation of GCDML into article metadata; or use of MIGS records to autogenerate GCDML tagged data for downstream use as an independent process. Final discussions focused on editorial issues. These included a review of instructions to authors, the proposed schedule for April, May and June issues of SIGS, and required resources. Immediate goals include greater automation of content processing by sequencing centers, drafting of a protocol describing the assembly of short genome reports, and finalizing the output formats of content. This latter point involves the conversion of edited manuscripts to HTML, PDF and XML instances that are needed to meet expectations and requirements.

Although mid-April was proposed as the date of publication for the first issue, a number of unresolved issues surfaced as we moved forward that required further consideration, including: stricter definition of reference types, refinement of the document structure of various articles and improvement of the underlying model used for short genome reports based on valuable suggestions received from peer-reviewers. Our immediate goal is to fill the editorial queue with sufficient content to sustain publication of the first three issues. Although we intend to publish on a monthly basis, it is possible that our first several issues may take longer to complete due to aspects of production that are still under development.

Other known production issues that cannot yet be addressed are linkage of bibliographies into the CrossRef and PubMed indices. For these tasks, we will need to acquire licenses for eXtyles and AntennaHouse. Some custom work will also be required to streamline the processing and transformation of XML files into custom-defined text-based documents. We have opted for this investment as it will provide SIGS with the opportunity to publish high quality content very soon after acceptance of an article, at minimal cost. The alternative route of using a service-bureau for this part of the production process, while attractive, would require much longer lead times and piecemeal production of articles to hold down costs.

Organizational issues were also discussed briefly. Key topics included scheduling of regular meetings (biannual) and editorial teleconferences (biweekly). Improved webcasting tools also need to be acquired prior to SIGS2. Legal incorporation of SIGS as a non-profit organization must also be pursued, either in conjunction with the GSC or as a separate entity to allow SIGS to fully function as an independent publisher. This will also allow SIGS/GSC to pursue funding opportunities in the US and EU as stand-alone organizations.
